# Signaling through Syk or CARD9 Mediates Species-Specific Anti-*Candida* Protection in Bone Marrow Chimeric Mice

**DOI:** 10.1128/mBio.01608-21

**Published:** 2021-08-31

**Authors:** Erik Zajta, Katalin Csonka, Adél Tóth, Laszló Tiszlavicz, Tamás Németh, Anita Orosz, Ádám Novák, Máté Csikós, Csaba Vágvölgyi, Attila Mócsai, Attila Gácser

**Affiliations:** a HCEMM-USZ Fungal Pathogens Research Group, Department of Microbiology, Faculty of Science and Informatics, University of Szeged, Szeged, Hungary; b Department of Pathology, University of Szeged, Szeged, Hungary; c Department of Physiology, Semmelweis University School of Medicine, Budapest, Hungary; d MTA-SZTE “Lendület” Mycobiome Research Group, University of Szeged, Szeged, Hungary; e Department of Rheumatology and Clinical Immunology, Semmelweis University, Budapest, Hungary; Universidade de Sao Paulo

**Keywords:** *Candida parapsilosis*, *Candida albicans*, Syk, CARD9, antifungal immunity, antifungal immune therapy, bone marrow chimera

## Abstract

The spleen tyrosine kinase (Syk) and the downstream adaptor protein CARD9 are crucial signaling molecules in antimicrobial immunity. Candida parapsilosis is an emerging fungal pathogen with a high incidence in neonates, while Candida albicans is the most common agent of candidiasis. While signaling through Syk/CARD9 promotes protective host mechanisms in response to C. albicans, its function in immunity against C. parapsilosis remains unclear. Here, we generated Syk^−/−^ and CARD9^−/−^ bone marrow chimeric mice to study the role of Syk/CARD9 signaling in immune responses to C. parapsilosis compared to C. albicans. We demonstrate various functions of this pathway (e.g., phagocytosis, phagosome acidification, and killing) in *Candida*-challenged, bone marrow-derived macrophages with differential involvement of Syk and CARD9 along with species-specific differences in cytokine production. We report that Syk^−/−^ or CARD9^−/−^ chimeras rapidly display high susceptibility to C. albicans, while C. parapsilosis infection exacerbates over a prolonged period in these animals. Thus, our results establish that Syk and CARD9 contribute to systemic resistance to C. parapsilosis and C. albicans differently. Additionally, we confirm prior studies but also detail new insights into the fundamental roles of both proteins in immunity against C. albicans. Our data further suggest that Syk has a more prominent influence on anti-*Candida* immunity than CARD9. Therefore, this study reinforces the Syk/CARD9 pathway as a potential target for anti-*Candida* immune therapy.

## INTRODUCTION

Advancements in the study of antifungal immunity have revealed various approaches to promote recovery from fungal diseases through the modulation of immune responses. Although adjunctive antifungal immune therapies signify a promising direction in aiding the efficiency of antimycotic drugs, several challenges hinder their development (1–3). One difficulty is that taxonomically close species may induce dissimilar immune responses.

Invasive *Candida* infections have a worldwide annual incidence of ∼700.000 (4), and they are associated with a mortality of ∼30 to 75% (5, 6). Although C. albicans is the most frequent agent, C. parapsilosis has a record of globally increasing incidence, and it poses a particular threat to neonates and patients on parenteral nutrition (6–9). The two species may induce different host responses. For instance, C. albicans favors the polarization of Th cells to the Th17 phenotype more efficiently than C. parapsilosis in cell culture (10). Also, unlike C. albicans, C. parapsilosis triggers prominent interleukin 27 (IL-27) production to suppress protective inflammatory processes in mice. Therefore, the notion of therapeutically blocking IL-27 signaling during C. parapsilosis infections has been raised, underlining the need to identify species-specific immune reactions (11). The differences between the immunogenicity of the two species arise partially from their nonidentical morphology, cell wall composition, and interaction with pattern recognition receptors (PRRs) (12).

A major function of the spleen tyrosine kinase (Syk) is to mediate signaling initiated by PRRs (e.g., Dectin-1) binding microbial structures (13, 14), especially fungal pathogen-associated molecular patterns (15–17). A major pathway proceeding through PKCδ and Vav proteins (18, 19) relies on the downstream adaptor caspase recruitment domain-containing protein 9 (CARD9), which functions as a component of supramolecular structures such as the CARD9-BCL10-MALT-1 or the CARD9-H-Ras-Ras-GRF1 complexes (20–22). Nevertheless, Syk and CARD9 can operate independently (23, 24). Upon fungal stimuli, the Syk/CARD9 pathway results in the activation of innate antifungal mechanisms that may later shape adaptive immunity (23, 25–33). Consequently, deficiency in Syk/CARD9 signaling increases susceptibility to invasive fungal infections in both humans and mice (21, 25, 26, 28, 33–36). For example, CARD9 mutations have been associated with chronic mucocutaneous candidiasis, meningoencephalitis, and colitis caused by C. albicans (26, 34, 35). Although multiple studies have underlined the pivotal role of Syk and CARD9 in immunity against C. albicans (21, 25, 26, 28, 33, 37), several findings are conferred only from chemical inhibition of Syk activity or the use of artificial yeast mimics (e.g., zymosan or heat-killed cells). In contrast, the only direct link between this pathway and C. parapsilosis-induced immune responses is the compromised IL-1β production of Syk-blocked THP-1 cells after fungal challenge (38).

In this study, we aimed to ascertain if Syk/CARD9 signaling is a prominent component of immunity to C. parapsilosis. We demonstrate that this pathway is involved in various immune responses evoked by C. parapsilosis in bone marrow-derived macrophages (BMDMs). Utilizing Syk- or CARD9-deficient bone marrow chimeric mice (referred to as Syk^−/−^ or CARD9^−/−^ chimeras and the respective wild-type controls as Wt_Syk_ and Wt_CARD9_ chimeras), we also provide evidence that this pathway confers protection in the setting of invasive C. parapsilosis infection, which becomes especially apparent in the late phase of the infection. As most experiments were carried out in comparison with live C. albicans, we confirm previous studies and provide new insights into the essential roles of Syk and CARD9 in immunity against this pathogen. Finally, we demonstrate that this signaling pathway is differentially involved in mediating protection against the two *Candida* species *in vivo*.

## RESULTS

### Syk and CARD9 mediate nuclear translocation of NF-κB p65 in C. parapsilosis*-*treated BMDMs.

**FIG 1:**
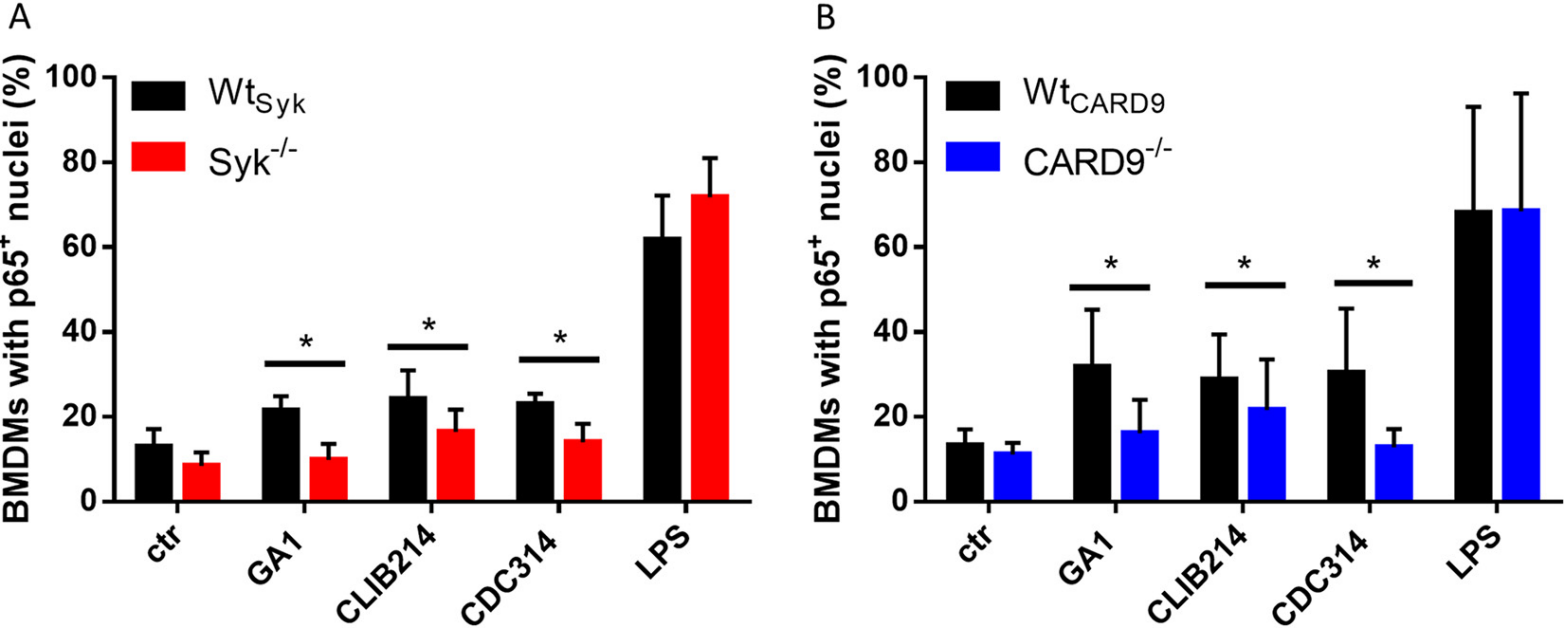
Nuclear translocation of NF-κB p65 in BMDMs upon C. parapsilosis infection. (A and B) BMDMs were treated with C. parapsilosis (strains GA1, CLIB214, and CDC317; MOI of 5:1) or LPS (1 μg/ml) for 90 min, or untreated control (ctr) cells were used. Nuclei were stained with DRAQ5 and NF-κB p65 with an Alexa Fluor 488-conjugated antibody. Cells were analyzed by imaging flow cytometry. Percentage of Wt_Syk_, Syk^−/−^ (A), Wt_CARD9_, and CARD9^−/−^ (B) BMDMs with p65^+^ nuclei are shown. Data represent the mean ± SD. Data are pooled from a minimum of 4 independent experiments. The paired Student's *t* test was applied. *, *P* < 0.05.

The classical activation of the NF-κB pathway through nuclear translocation of the p65 subunit is a major component of antifungal immunity (39–41). Recently, C. parapsilosis was shown to trigger this translocation in THP-1 cells (42). However, it is unclear whether Syk or CARD9 controls this process. Therefore, we implemented immune staining of p65 to monitor its translocation in C. parapsilosis-infected (strains GA1, CLIB214, and CDC317) BMDMs cultured from Wt_Syk_, Syk^−/−^, Wt_CARD9_, and CARD9^−/−^ chimeras. We found a decreased proportion of Syk^−/−^ or CARD9^−/−^ macrophages with p65^+^ nuclei compared to Wt_Syk_ or Wt_CARD9_ cells, respectively, in case of all strains (mean ± SD of percent decrease compared to wild-type [WT] control cells; *Syk^−/−^*, GA1, 51.06 ± 23.40; CLIB214, 31.89 ± 11.35; CDC314, 39.33 ± 15.40; *CARD9^−/−^*, GA1, 44.75 ± 26.56; CLIB214, 26.58 ± 22.29; CDC314, 49.89 ± 26.03) (Fig. 1). Lipopolysaccharide (LPS)-induced NF-κB activation served as positive control, with over 60% of BMDMs displaying p65 translocation regardless of genetic background (Fig. 1). These findings suggest that activation of NF-κB in C. parapsilosis-infected BMDMs is under the influence of the Syk/CARD9 pathway.

### The Syk/CARD9 pathway differentially controls cytokine production of BMDMs in response to C. parapsilosis and C. albicans.

The involvement of Syk and CARD9 in NF-κB regulation suggested that the consequent cytokine production would also depend on these proteins during C. parapsilosis infection. Therefore, we assessed the cytokine response of Wt_Syk_, Syk^−/−^, Wt_CARD9,_ and CARD9^−/−^ BMDMs following fungal stimuli.

First, we used proteome profiler membranes to identify cytokines in cell culture supernatants collected from BMDMs challenged with the GA1 strain. In the case of Wt_Syk_ and Wt_CARD9_ BMDMs, tumor necrosis factor alpha (TNF-α), and chemokines KC (CXCL1), MIP-1α (CCL3), and MIP-2 (CXCL2) were detected (Fig. S1 in the supplemental material). Compared to the WT BMDMs, the TNF-α yield from Syk^−/−^ or CARD9^−/−^ cells was diminished after the fungal challenge. While the chemokine secretion of Syk^−/−^ BMDMs appeared intact (Fig. S1A), CARD9^−/−^ cells secreted hardly any or no detectable KC, MIP-1α, and MIP-2 compared to the corresponding WT cells (Fig. S1B). These cytokines were not detected in the supernatants of uninfected control BMDMs from any genetic background (data not shown).

10.1128/mBio.01608-21.1 FIG S1 Proteome profiler results. (A and B) BMDMs were treated with C. parapsilosis (strain GA1; MOI of 5:1) for 24 h. Pooled supernatants from at least 3 independent experiments were analyzed for cytokines by the R&D Systems Proteome Profiler array mouse cytokine array panel A kit. Chemiluminescence intensity indicating TNF-α, KC, MIP-1α, and MIP-2 production of Wt_Syk_, Syk^−/−^ (A), Wt_CARD9_, and CARD9^−/−^ BMDMs (B) is shown. Download FIG S1, TIF file, 0.2 MB. Copyright © 2021 Zajta et al. 2021 Zajta et al. https://creativecommons.org/licenses/by/4.0/ This content is distributed under the terms of the Creative Commons Attribution 4.0 International license.

**FIG 2:**
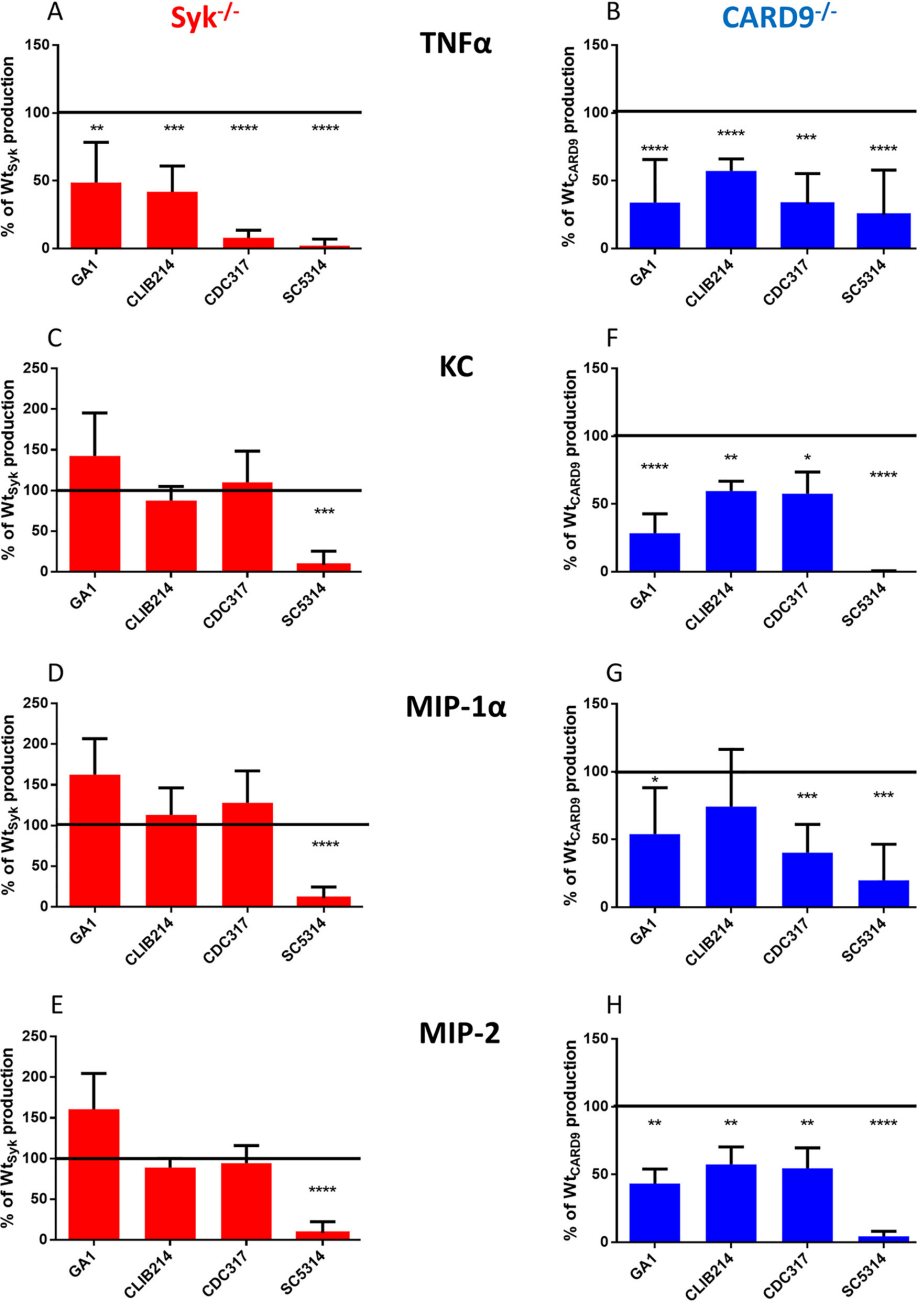
Cytokine production of BMDMs upon C. parapsilosis or C. albicans infection. (A to H) Wt_Syk_, Syk^−/−^ (A, C, D, and E), Wt_CARD9_, and CARD9^−/−^ (B, F, G, and H) BMDMs were treated with C. parapsilosis (strains GA1, CLIB214 and CDC317; MOI of 5:1) or C. albicans (strain SC5314; MOI of 1:25) for 24 h. Cell culture supernatants were analyzed for TNF-α (A and B), KC (C and F), MIP-1α (D and G), and MIP-2 (E and H) by ELISA. Percentages of WT control values are shown here, while absolute concentrations are listed in Table S1 in the supplemental material. Data represent the mean ± SD. Data are pooled from a minimum of 4 independent experiments. The paired Student's *t* test was applied. *, *P* < 0.05; **, *P* < 0.01; ***, *P* < 0.001; ****, *P* < 0.0001. Cytokine production measured by proteome profiler membranes is shown in Fig. S1.

Next, we sought to test these observations with enzyme-linked immunosorbent assay (ELISA), including multiple C. parapsilosis strains (GA1, CLIB214, and CDC317) and the SC5314 C. albicans strain as reference (Fig. 2 and Table S1). The TNF-α production of C. parapsilosis- or C. albicans-treated Syk^−/−^ or CARD9^−/−^ macrophages was lower than that of Wt_Syk_ and Wt_CARD9_ cells by >42% (Fig. 2A and B). While the chemokine yield of C. albicans-stimulated Syk^−/−^ macrophages dropped by ∼90% in comparison to Wt_Syk_ BMDMs, the KC, MIP-1α, and MIP-2 expression of C. parapsilosis-treated Syk^−/−^ BMDMs did not decrease (Fig. 2C, D, and E). In contrast, we found a decrease of at least 25% in the chemokine production of *Candida*-infected CARD9^−/−^ BMDMs (with the exception of CLIB214 for MIP-1α production) (Fig. 2F, G, and H). Notably, the dependence of the production of any studied cytokines on either Syk or CARD9 was greatest in the case of the C. albicans strain (Fig. 2A to H).

10.1128/mBio.01608-21.5 TABLE S1 Absolute cytokine concentrations (pg/ml) ± SD measured by ELISA from cell culture supernatants of BMDMs treated with C. parapsilosis (strains GA1, CLIB214, or CDC317; MOI of 5:1; 24 h) or C. albicans (strain SC5314; MOI of 1:25; 24 h). Data are pooled from a minimum of 4 independent experiments. ctr, control, Cp−, C. parapsilosis; Ca−, C. albicans. Download Table S1, PDF file, 0.3 MB. Copyright © 2021 Zajta et al. 2021 Zajta et al. https://creativecommons.org/licenses/by/4.0/ This content is distributed under the terms of the Creative Commons Attribution 4.0 International license.

Taken together, we concluded that both Syk and CARD9 participate in the regulation of cytokine production of BMDMs upon both C. parapsilosis and C. albicans infection, but the involvement of Syk differs between the two *Candida* species.

### Syk, but not CARD9, promotes phagocytosis of C. parapsilosis and C. albicans by BMDMs and subsequent phagosome acidification.

PRRs exploiting the Syk/CARD9 pathway have been indicated as mediators of phagocytosing fungal elements (15, 43–45). Additionally, heat-killed *Candida* cells were shown to be ingested in a Syk-dependent manner by neutrophils (46). As C. parapsilosis and C. albicans cells contain motives recognized by Syk/CARD9-dependent receptors, we hypothesized that internalization of the live form of these yeasts by macrophages was also regulated by this pathway.

**FIG 3:**
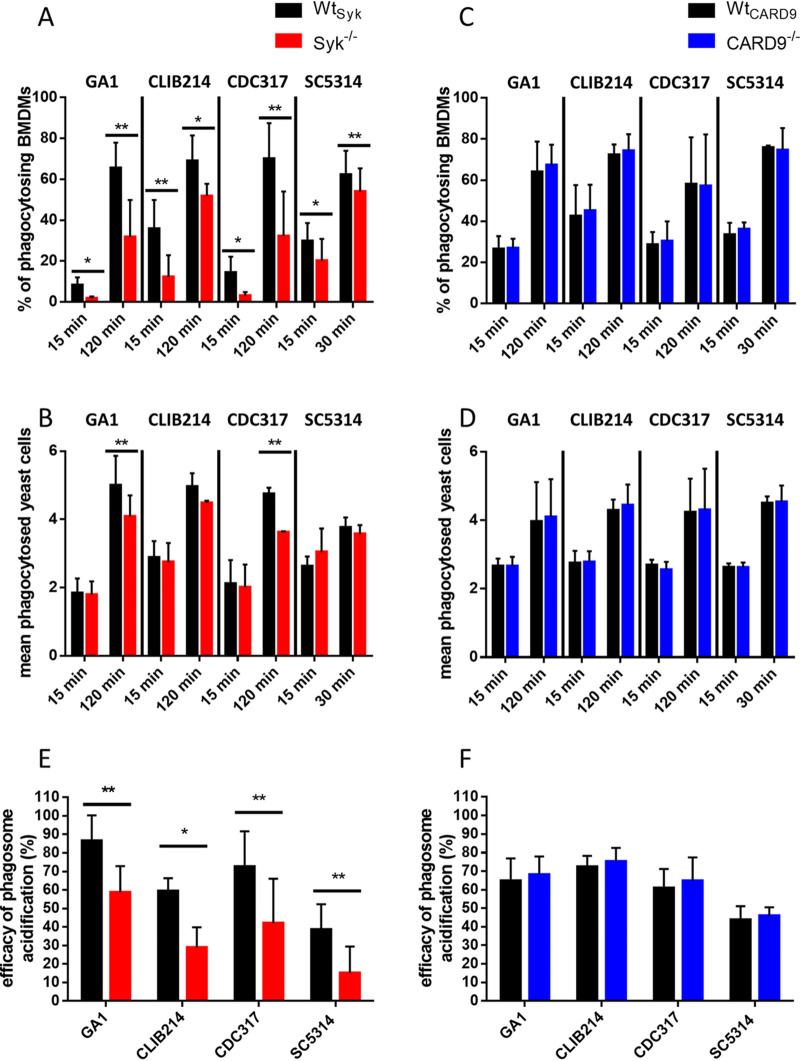
BMDM phagocytosis efficiency and phagosome acidification upon C. parapsilosis or C. albicans infection. (A to D) Wt_Syk_, Syk^−/−^ (A and B), Wt_CARD9_, and CARD9^−/−^ (C and D) BMDMs were treated with fluorescent (Alexa Fluor 488/GFP) C. parapsilosis (strains GA1, CLIB214, and CDC317; MOI of 5:1) for 15 min and 2 h or with C. albicans (strain SC5314; MOI of 5:1) for 15 and 30 min. Phagocytosis was assessed by imaging flow cytometry. Percentages of phagocytosis-positive macrophages (A and C) and the mean number of ingested yeasts per macrophage (B and D) within the phagocytosing population are shown. (E and F) Wt_Syk_, Syk^−/−^ (E), Wt_CARD9_, and CARD9^−/−^ (F) BMDMs were treated with double-labeled (Alexa Fluor 488/GFP plus pHrodo Red) C. parapsilosis or C. albicans (MOI of 5:1) for 15 min. Efficacy of phagosome acidification was determined by imaging flow cytometry. Data represent the mean ± SD. Data are pooled from a minimum of 3 independent experiments. The paired Student's *t* test was applied. *, *P* < 0.05; **, *P* < 0.01.

Therefore, we infected BMDMs with fluorescently labeled (Alexa Fluor 488 or green fluorescent protein [GFP]) C. parapsilosis or C. albicans cells to monitor their uptake using imaging flow cytometry. Early (15 min) and late (120 and 30 min for C. parapsilosis and C. albicans, respectively) experimental time points were selected. The proportion of phagocytosis-positive Wt_Syk_ macrophages significantly exceeded that of Syk^−/−^ cells regardless of *Candida* species or incubation time (Fig. 3A). Furthermore, the average number of ingested yeast cells/BMDM within the phagocytosing population was significantly higher in Wt_Syk_ cells than in Syk^−/−^ macrophages in the case of the GA1 and CDC317 C. parapsilosis strains at the late time point. A trend of nonsignificant decrease of this parameter was also detectable in CLIB214- or SC5314-treated Syk^−/−^ BMDMs at this time point (Fig. 3B). In contrast, CARD9^−/−^ cells phagocytosed both C. parapsilosis and C. albicans as effectively as their Wt_CARD9_ counterparts (Fig. 3C and D). Thus, we concluded that Syk, but not CARD9, contributes to the ingestion of both live C. parapsilosis and C. albicans by BMDMs.

As Syk was involved in the acidification of phagosomes containing heat-killed C. albicans in RAW cells (31, 47), our next step was testing if phagosome acidification following the phagocytosis of live C. parapsilosis and C. albicans also relies on this pathway in BMDMs. To this end, we double labeled C. parapsilosis and C. albicans cells with Alexa Fluor 488 or GFP together with pHrodo Red, a pH-sensitive dye that gains fluorescence upon acidification of phagosomes. Following coincubation with BMDMs, the Alexa Fluor 488^+^/GFP^+^ and pHrodo Red^+^ macrophage populations were examined to determine the efficiency of phagosome acidification. Relative to Wt_Syk_ cells, phagosome acidification was less efficient in Syk^−/−^ cells after coincubation with any of the applied strains (Fig. 3E). In contrast, phagosome acidification was unaltered in CARD9^−/−^ BMDMs (Fig. 3F). Therefore, the acidification of phagosomes containing C. parapsilosis or C. albicans is mediated by Syk, but not CARD9, in BMDMs.

### Syk, but not CARD9, plays a role in the killing of C. parapsilosis by BMDMs.

Previous studies revealed that the Syk/CARD9 signal transduction may modulate the candidacidal activity of phagocytes or intracellular replication of *Candida* cells within innate immune cells (15, 26, 36, 48–50). Also, our current findings show that the phagocytosis of C. parapsilosis by murine macrophages and the subsequent phagosome acidification is regulated by Syk. Thus, we speculated that Syk^−/−^, but not CARD9^−/−^, macrophages would be defective in the elimination of C. parapsilosis cells.

**FIG 4:**
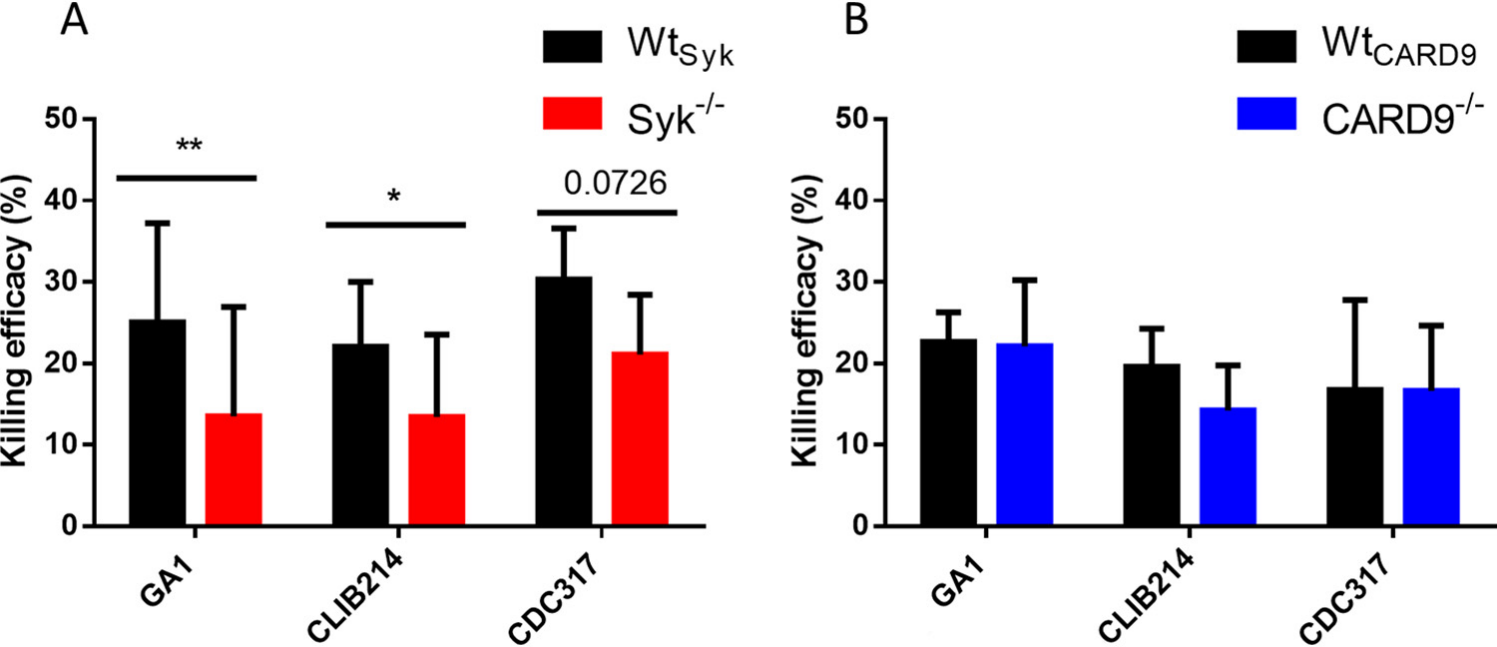
Killing efficacy of macrophages upon C. parapsilosis infection. (A and B) Wt_Syk_, Syk^−/−^ (A), Wt_CARD9_, and CARD9^−/−^ (B) BMDMs were treated with C. parapsilosis (strains GA1, CLIB214, and CDC317; MOI of 5:1) for 3 h. Killing efficacy was determined by CFU counting. Data represent the mean ± SD. Data are pooled from a minimum of 3 independent experiments. The paired Student's *t* test was applied. *, *P* < 0.05; **, *P* < 0.01.

Therefore, we compared the killing efficacy of WT and mutant macrophages. Truly, Syk^−/−^ BMDMs displayed diminished killing efficiency against C. parapsilosis, although the reduction only followed a nonsignificant (*P* = 0.0726) trend in the case of the CDC317 strain (Fig. 4A). As expected, CARD9^−/−^ BMDMs eliminated C. parapsilosis similarly to Wt_CARD9_ BMDMs (Fig. 4B).

Therefore, killing of C. parapsilosis is regulated by Syk, but not CARD9, in BMDMs.

### Susceptibility of Syk^−/−^ or CARD9^−/−^ bone marrow chimeric mice to C. parapsilosis and C. albicans follows dissimilar patterns.

**FIG 5:**
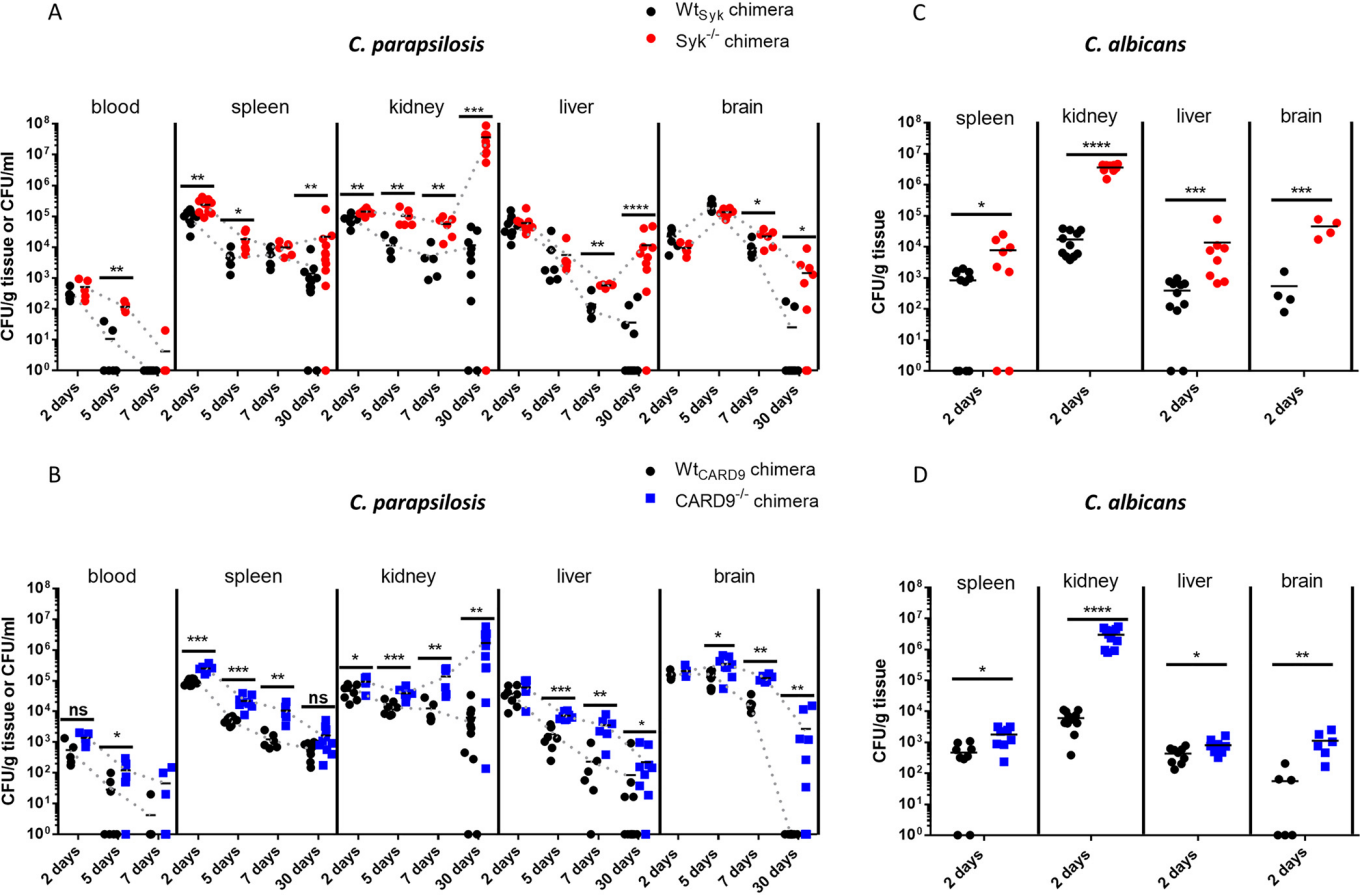
Susceptibility to systemic C. parapsilosis and C. albicans infection. (A to D) Wt_Syk_, Syk^−/−^ (A and C), Wt_CARD9_, and CARD9^−/−^ (B and D) bone marrow chimeric mice were infected i.v. with C. parapsilosis GA1 (2 × 10^7^ yeast cells/mouse) (A and B) or C. albicans SC5314 (10^5^ yeast cells/mouse) (C and D). Animals were euthanized at 2, 5, 7, or 30 (A and B) dpi. Blood or spleen, kidney, liver, and brain homogenates were plated on YPD plates, and CFU were determined 2 days later. Data are pooled from at least 2 independent experiments. The Mann-Whitney test was applied. *, *P* < 0.05; **, *P* < 0.01; ***, *P* < 0.001; ****, *P* < 0.0001; ns, not significant.

As our *in vitro* studies revealed the participation of Syk/CARD9 signaling in the immune recognition of C. parapsilosis, we hypothesized that the absence of either Syk or CARD9 in hematopoietic cells would compromise host immunity upon systemic C. parapsilosis infections. To test this, we challenged Syk^−/−^, CARD9^−/−^, and their corresponding WT control bone marrow chimeric mice with the C. parapsilosis GA1 strain via intravenous (i.v.) injection. C. albicans SC5314 was used as a reference strain. Fungal burden in C. parapsilosis-treated animals was evaluated 2, 5, 7, and 30 days postinfection (dpi). As injection of Syk^−/−^ or CARD9^−/−^ chimeras with C. albicans was lethal by 4 dpi (data not shown), fungal burden was determined at 2 dpi. At this time, no significant difference was detected between the C. parapsilosis-challenged Wt_Syk_ and Syk^−/−^ or Wt_CARD9_ and CARD9^−/−^ chimeras in terms of blood, liver, and brain tissue colonization (Fig. 5A and B). However, fungal burden in the spleen and kidneys of Syk^−/−^ chimeras was ∼2.2-fold and ∼1.7-fold higher, respectively, than in Wt_Syk_ animals (Fig. 5A). Likewise, ∼2.7-fold and ∼2-fold higher colonization was detected in the spleen and kidneys of C. parapsilosis-infected CARD9^−/−^ chimeras compared to their WT counterparts (Fig. 5B). At later time intervals, C. parapsilosis colonization of all examined organs of the Syk^−/−^ or CARD9^−/−^ chimeras exceeded that of the respective WT chimeras in most cases (Fig. 5A and B). The difference was most apparent at 30 dpi where fungal burden was over 58-fold (brain), 310-fold (liver), and 3,100-fold (kidneys) higher in Syk^−/−^ chimeras than in Wt_Syk_ chimeras (Fig. 5A). At this time, over 270 times higher colonization was determined in the kidneys of CARD9^−/−^ chimeric mice than Wt_CARD9_ controls (Fig. 5B). Also, while C. parapsilosis was cleared from the brain of all Wt_CARD9_ chimeras, the mean fungal burden in this organ was 2,700 CFU/g in the CARD9^−/−^ genotype (Fig. 5B). Additionally, kinetic patterns suggested that the tendency toward an initial clearance was reversed by 30 dpi in the spleen, liver, and kidneys of Syk^−/−^ and the kidneys of CARD9^−/−^ chimeras (Fig. 5A and B). Moreover, 2 of the C. parapsilosis-infected Syk^−/−^ chimeras were euthanized before day 30 due to deteriorated health conditions. The kidneys of these animals were severely deformed by sizable abscesses full of yeast cells (Fig. S2). However, macroscopic renal abscesses were not seen on the kidneys of any other C. parapsilosis-infected mice.

10.1128/mBio.01608-21.2 FIG S2 Kidneys of a Syk^−/−^ bone marrow chimera infected with C. parapsilosis and euthanized at 26 dpi. Infected (2 × 10^7^ yeast cells/mouse) kidneys isolated from the animal (A) and the PAS-stained section of one of the kidneys (B) are shown. Scale, 100 μm. White arrows point to fungal abscesses; black arrows point to areas extensively occupied by yeast cells. Download FIG S2, EPS file, 1.5 MB. Copyright © 2021 Zajta et al. 2021 Zajta et al. https://creativecommons.org/licenses/by/4.0/ This content is distributed under the terms of the Creative Commons Attribution 4.0 International license.

In contrast to the C. parapsilosis challenge at 2 dpi, significantly higher fungal burdens were detected in all inspected organs of C. albicans-treated Syk^−/−^ or CARD9^−/−^ chimeras than the corresponding WT mice (Fig. 5C and D). The difference was most prominent in the kidneys (>200-fold), which also manifested in small abscesses in both the Syk^−/−^ and CARD9^−/−^ backgrounds (Fig. S3A and B). Nonetheless, no C. albicans colonies were recovered from the blood in any genetic background (data not shown).

10.1128/mBio.01608-21.3 FIG S3 Morphology of kidneys after C. parapsilosis and C. albicans infection at 2 dpi. Wt_Syk_, Syk^−/−^, (A), Wt_CARD9_, and CARD9^−/−^ (B) bone marrow chimeric mice were infected i.v. with C. parapsilosis GA1 (2 × 10^7^ yeast cells/mouse) or C. albicans SC5314 (10^5^ yeast cells/mouse). Kidneys collected from bone marrow chimeric mice at 2 dpi are shown. White arrows point to small fungal abscesses. Download FIG S3, TIF file, 2.0 MB. Copyright © 2021 Zajta et al. 2021 Zajta et al. https://creativecommons.org/licenses/by/4.0/ This content is distributed under the terms of the Creative Commons Attribution 4.0 International license.

**FIG 6:**
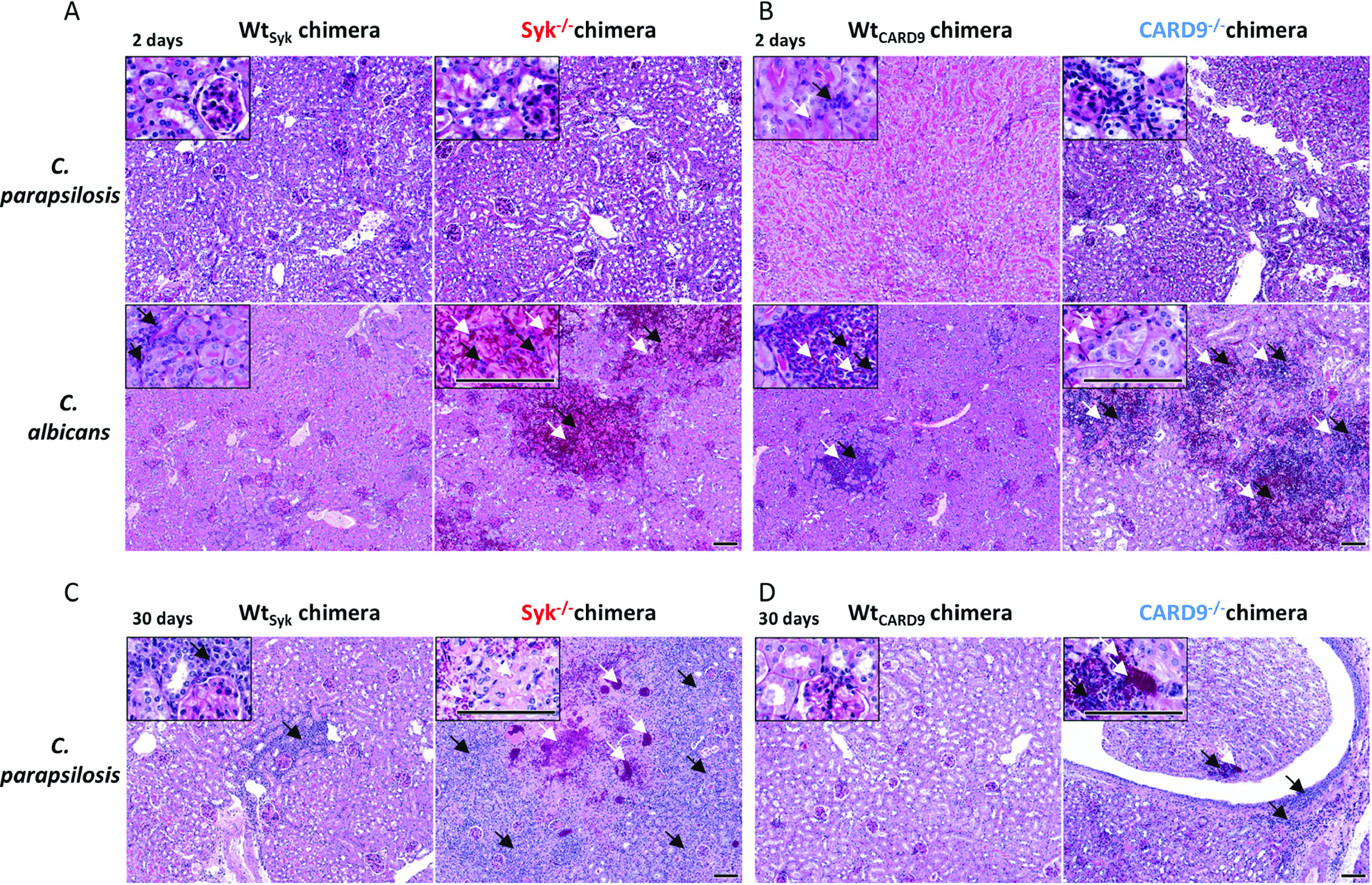
Histopathology of kidneys during C. parapsilosis and C. albicans infection. Wt_Syk_, Syk^−/−^(A and C) Wt_CARD9_, and CARD9^−/−^ (B and D) bone marrow chimeric mice were infected i.v. with C. parapsilosis GA1 (2 × 10^7^ yeast cells/mouse) or C. albicans SC5314 (10^5^ yeast cells/mouse) or mock infected with PBS (see Fig. S4A) and euthanized at 2 (A and B) or 30 (C and D) dpi. PAS-stained histological sections were prepared to monitor fungal elements (white arrows) and leukocyte infiltrates (black arrows). Scale, 100 μm. Kidney morphology of C. parapsilosis- or C. albicans-infected mice at 2 dpi are depicted in Fig. S2, while pathological features of a C. parapsilosis Syk^−/−^ chimera euthanized at 26 dpi due to severe health conditions are shown in Fig. S3.

As kidneys are the main target organs of systemic candidiasis in mice (51, 52) and our CFU data indicated marked differences in this organ between the Syk^−/−^ or CARD9^−/−^ and WT chimeras, we examined the presence of fungal elements and associated leukocyte infiltrates in the kidneys using periodic acid-Schiff (PAS)-stained histological tissue sections at 2 and 30 dpi (Fig. 6 and Fig. S4A). At 2 dpi, yeast cells or leukocytes were only sporadically detectable in C. parapsilosis-treated mice of any genetic background (Fig. 6A and B). While C. albicans hyphae/yeast cells and immunocyte clusters were sometimes observed in the kidneys of WT chimeras, extensive patches occupied by fungal structures mingling with infiltrating leukocytes and necrotic tissues were conspicuous in the kidneys of Syk^−/−^ or CARD9^−/−^ chimeras at 2 dpi (Fig. 6A and B). At day 30, yeast cells or frequent leukocyte infiltrates were not characteristic of C. parapsilosis-infected WT chimeras but were observed in Syk^−/−^ and CARD9^−/−^ chimeras with detectable tissue necrosis in Syk^−/−^ chimeras (Fig. 6C and D). Nevertheless, while immune cells and C. parapsilosis yeast clusters were always present in the kidneys of Syk^−/−^ chimeras, these traits were not always detected in CARD9^−/−^ chimeras.

10.1128/mBio.01608-21.4 FIG S4 Histological kidney sections of mock-infected control bone marrow chimeric mice. (A) Wt_Syk_, Syk^−/−^, Wt_CARD9_, and CARD9^−/−^ bone marrow chimeric mice were injected with 100 μl PBS and euthanized at 2 or 30 dpi, and kidneys were collected for histological preparations. PAS or MPO staining was applied. (B) Mice were treated similarly and euthanized at 30 dpi, and kidneys were collected for histological preparations. MPO, CD3, or CD68 staining was applied. Scale, 100 μm. Download FIG S4, EPS file, 1.9 MB. Copyright © 2021 Zajta et al. 2021 Zajta et al. https://creativecommons.org/licenses/by/4.0/ This content is distributed under the terms of the Creative Commons Attribution 4.0 International license.

Therefore, the absence of Syk or CARD9 in the hematopoietic cell population led to increased susceptibility to C. parapsilosis invasive infection. However, unlike in the case of C. albicans, massive fungal growth occurred only at a later stage of infection.

### Rapid inflammation in C. albicans-infected, but not in C. parapsilosis-infected, kidneys of Syk^−/−^ or CARD9^−/−^ bone marrow chimeric mice.

**FIG 7:**
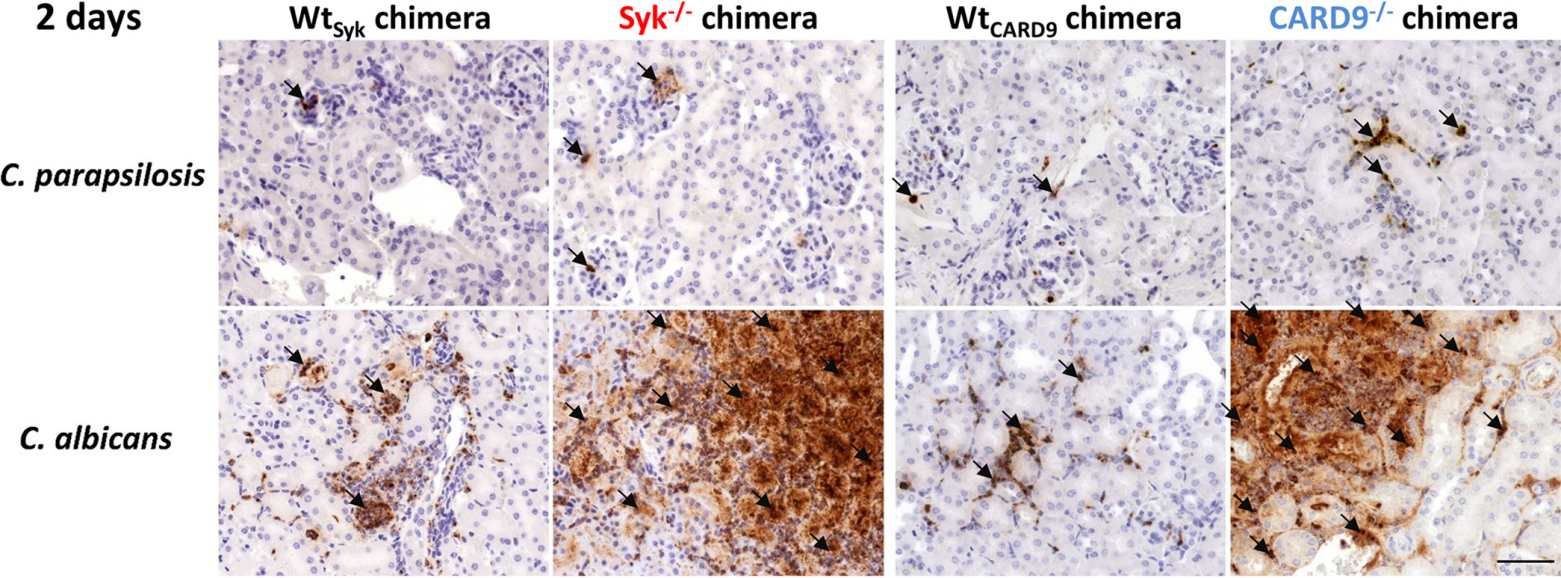
Presence of MPO in histological kidney sections at 2 dpi. Wt_Syk_, Syk^−/−^, Wt_CARD9_, and CARD9^−/−^ bone marrow chimeric mice were infected i.v. with C. parapsilosis (2 × 10^7^ yeast cells/mouse) or C. albicans (10^5^ yeast cells/mouse) or mock infected with PBS (see Fig. S4), and animals were euthanized at 2 dpi. Immune staining for MPO (brown) was implemented to detect neutrophil granulocytes. Black arrows indicate positivity for MPO staining. Scale, 100 μm.

**FIG 8:**
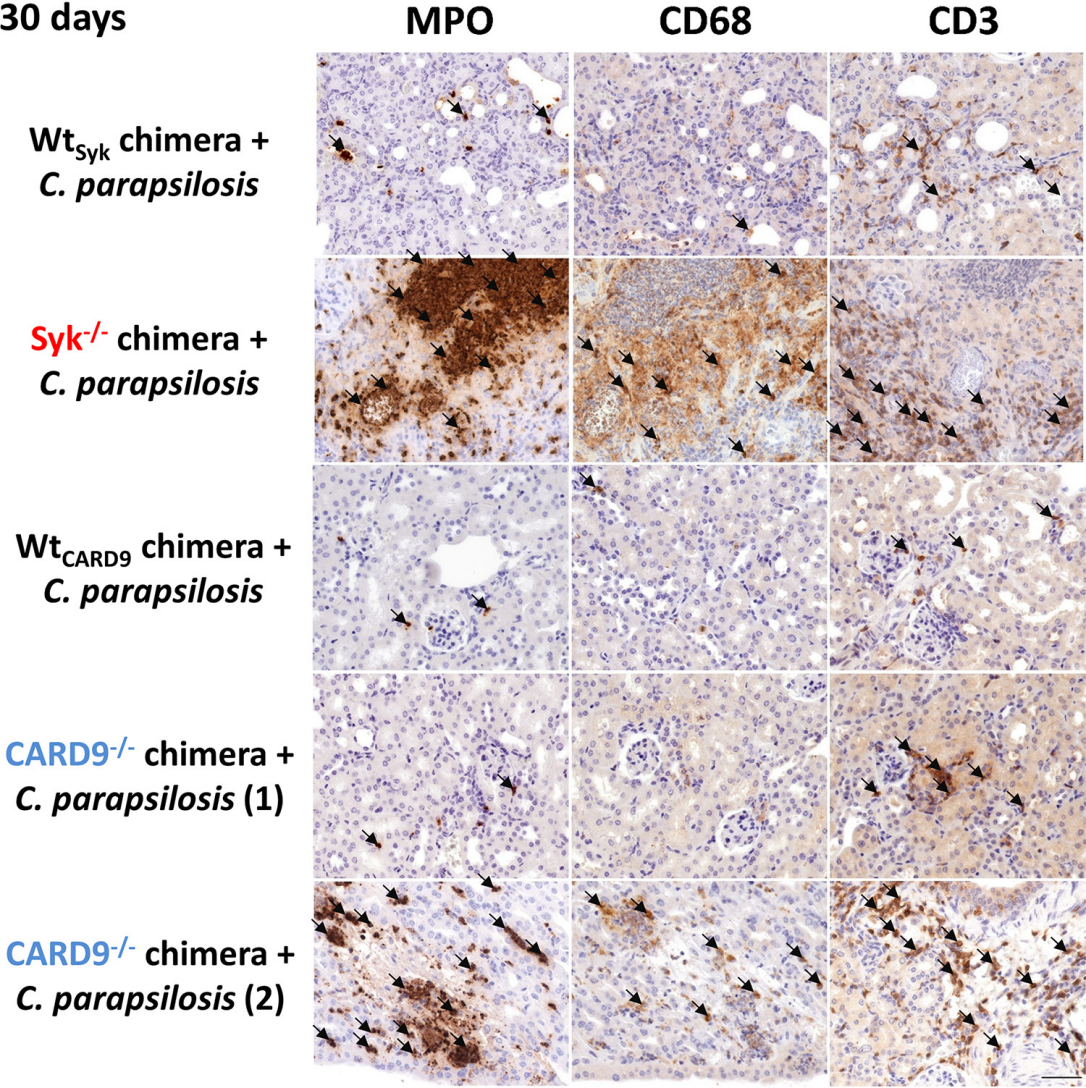
Presence of MPO and CD68^+^ or CD3^+^ cells in histological kidney sections at 30 dpi. Wt_Syk_, Syk^−/−^, Wt_CARD9_, and CARD9^−/−^ bone marrow chimeric mice were infected i.v. with C. parapsilosis (2 × 10^7^ yeast cells/mouse) or C. albicans (10^5^ yeast cells/mouse) or mock infected with PBS (see Fig. S4B), and animals were euthanized at 30 dpi. Immune staining for MPO, CD68, and CD3 (brown) was implemented to detect neutrophil granulocytes, macrophages, and T cells, respectively. Note that 2 sets of microphotographs are shown for two CARD9^−/−^ chimeras. Black arrows indicate positivity for MPO, CD68, or CD3 staining. Scale, 100 μm.

**FIG 9:**
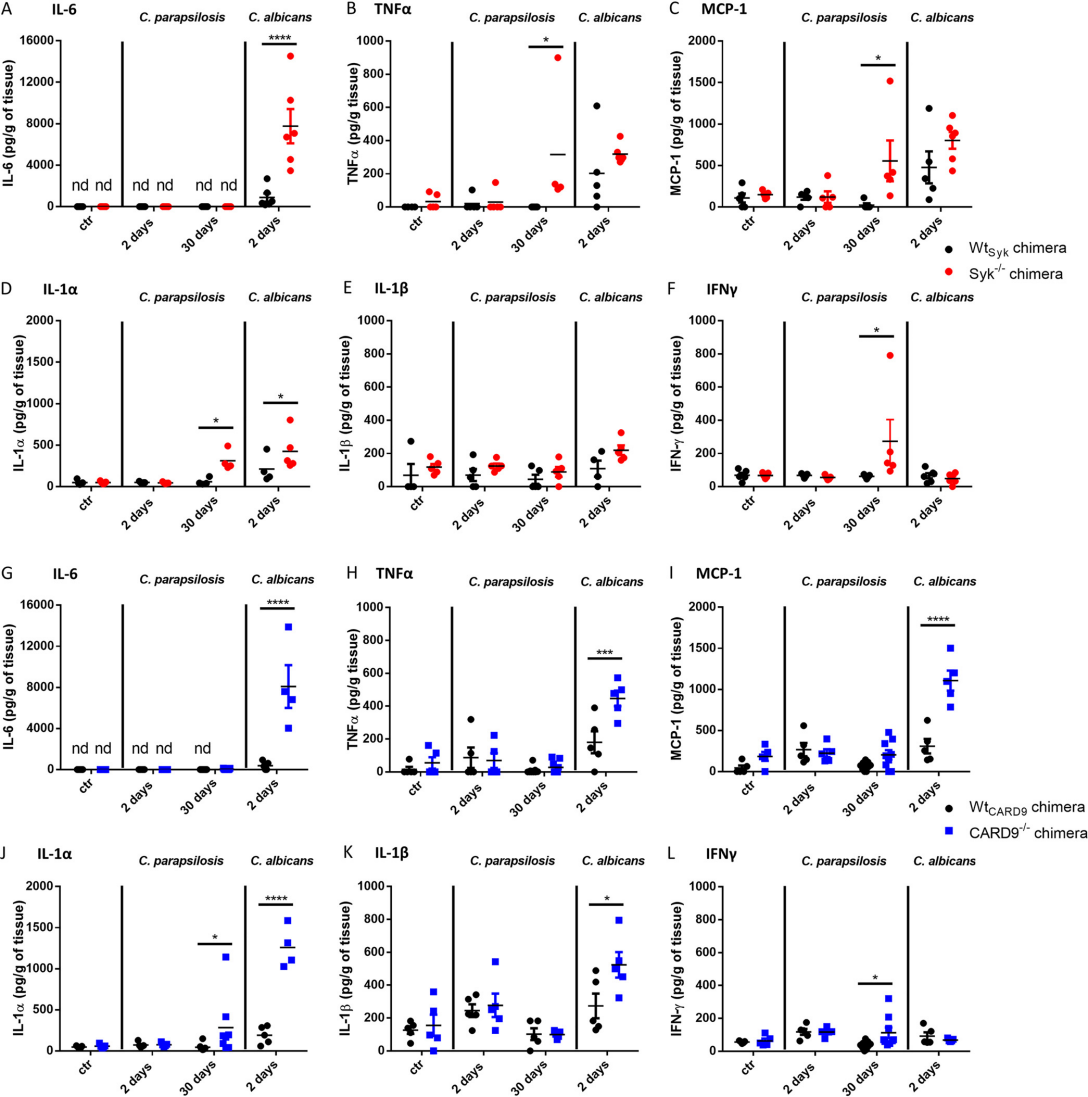
Cytokine production in kidneys following systemic infection with C. parapsilosis and C. albicans. (A to F) Wt_Syk_ and Syk^−/−^ bone marrow chimeras were infected i.v. with C. parapsilosis (2 × 10^7^ yeast cells/mouse) or C. albicans (10^5^ yeast cells/mouse) or mock infected with PBS (ctr), and animals were euthanized at 2 or 30 dpi. Cytokines were measured from kidney homogenates with ELISA. (G to L) Wt_CARD9_ and CARD9^−/−^ bone marrow chimeras were infected, and kidney homogenates were analyzed for cytokines similarly to panels A to F. Data are pooled from at least 2 independent experiments. Two-way ANOVA was applied. *, *P* < 0.05; ***, *P* < 0.001; ****, *P* < 0.0001.

As leukocyte infiltrates were plentiful in the kidneys of C. albicans-infected, but not C. parapsilosis-infected, Syk^−/−^ or CARD9^−/−^ chimeras at 2 dpi, we aimed to further characterize this inflammatory response in this organ. We found that mainly neutrophilic granulocytes occupied these areas as implied by expansive myeloperoxidase (MPO)-positive staining localized to infected sites (Fig. 7 and Fig. S4A). As noted, however, leukocyte infiltrates were present in the kidneys of C. parapsilosis-infected Syk^−/−^ or CARD9^−/−^ chimeras at 30 dpi. At this time, we found that neutrophils localized to infected sites were abundant in the kidneys of both mutant chimeras with CD68^+^ macrophages and CD3^+^ T cells also recruited to the tissues. However, these cell populations were not as consistent in CARD9^−/−^ chimeras as in Syk^−/−^ chimeras (Fig. 8B and Fig. S4B). At 2 dpi, inflammation was also detected by ELISA in the kidneys of C. albicans-infected Syk^−/−^ or CARD9^−/−^ chimeras, as kidney homogenates demonstrated an induction of IL-6, IL-1α, IL-1-β, TNF-α, and monocyte chemoattractant protein 1 (MCP-1) production, although only IL-6 and IL-1-α concentrations were significantly higher in the Syk^−/−^ than in the Wt_Syk_ background (Fig. 9A, D, G, H, I, J, and K). In contrast, C. parapsilosis did not induce prominent production of these proinflammatory cytokines in any genetic background at 2 dpi (Fig. 9). At 30 dpi, however, inflammation in the kidneys of C. parapsilosis-challenged Syk^−/−^ or CARD9^−/−^ chimeras was also confirmed by ELISA. While the amounts of TNF-α, MCP-1, IL-1α, and IFN-γ were higher in Syk^−/−^ than in Wt_Syk_ chimeric animals, only increased IL-1α and IFN-γ responses were observed in C. parapsilosis-infected CARD9^−/−^ chimeras (Fig. 9B, C, D, F, J, L).

Taken together, these data indicate that, while the absence of Syk or CARD9 in the hematopoietic cell population leads to an early, intense inflammatory response to C. albicans infection, C. parapsilosis triggers a mostly delayed Syk-dependent and moderately CARD9-dependent response in the kidneys of chimeric mice.

## DISCUSSION

In this study, we used bone marrow chimeric mice to define the involvement of Syk and CARD9 in immune responses to C. parapsilosis with comparisons to C. albicans. As was previously reported about C. albicans (23), we now demonstrated that both signaling proteins regulate the activation of NF-κB in C. parapsilosis-infected BMDMs. Intriguingly, C. glabrata led to Syk phosphorylation without NF-κB in macrophage-like cells (53). Therefore, C. parapsilosis resembles C. albicans rather than C. glabrata in regulating this transcriptional factor via the Syk/CARD9 pathway. Fitting with the role of NF-κB in governing cytokine production (17, 39, 40, 54), we established that the cytokine response of BMDMs to C. parapsilosis is influenced by Syk/CARD9 signaling. Furthermore, we showed that Syk controls cytokine production of C. albicans-challenged BMDMs as already described in Syk^−/−^ or SHP-2^−/−^ dendritic cells (DCs) (25, 33) and various CARD9^−/−^ cell types (21, 26, 28, 49). Notably, we found intact chemokine secretion in C. parapsilosis-stimulated, but not in C. albicans-stimulated, Syk^−/−^ BMDMs, suggesting species-specific differences in terms of Syk activation. Another interesting finding of our study was the CARD9-dependent, but Syk-independent, chemokine synthesis of BMDMs stimulated with C. parapsilosis. Similarly, a CARD9-dependent, but Syk-independent, mechanism was previously described in BMDMs challenged with C. albicans hyphae (23). Although Toll-like receptors (TLRs) (55, 56) and nucleotide-binding oligomerization domain 2 (NOD2) (22, 57) were proposed to signal through CARD9, future investigations are required to elucidate Syk-independent signaling through CARD9 in response to fungal stimuli.

Although multiple studies have covered the potential function of Syk and CARD9 in the phagocytosis of zymosan (25, 28, 29, 32, 58) or “yeast particles” (30), the direct role of Syk in leukocytes or CARD9 in macrophages in ingesting live C. parapsilosis or C. albicans cells has not been experimentally addressed to our knowledge. Our present results revealed that BMDMs phagocytose both species in a Syk-dependent, yet CARD9-independent, manner. Inhibition of Syk also hindered the phagocytosis of heat-killed C. albicans, C. glabrata, and C. auris by neutrophils (46). Our data are in line with the CARD9-independent internalization of C. albicans by human neutrophils and monocytes (26, 49). Similarly, we found that phagosome acidification in BMDMs challenged with either C. parapsilosis or C. albicans was regulated by Syk, but not CARD9. This supports the Syk-dependent phagosome acidification observed in RAW cells treated with heat-killed C. albicans by reference 31 and that Syk, but not CARD9, was required for proper maturation of C. albicans-containing phagosomes in macrophages (47). Previous research also showed that neutrophils kill C. albicans in a Syk-dependent manner (15, 46), while CARD9 was dispensable in this activity in murine neutrophils (50). Likewise, elimination of C. parapsilosis by BMDMs relied on Syk, but not CARD9, in our setting. Interestingly, human cells seem to differ from murine cells in that they can exploit CARD9 to eliminate C. albicans (15, 26, 49).

Several studies reported that deficiency in Syk (33) or CARD9 (21, 26, 28, 36) in mice leads to severe susceptibility to C. albicans (21, 26, 28, 33) or C. tropicalis (36) infections. Our invasive candidiasis experiments now revealed that Syk and CARD9 in hematopoietic cells contribute to systemic resistance to C. parapsilosis. Furthermore, we reaffirmed the crucial and almost immediate role of this pathway in the case of C. albicans. Although excessive inflammation (cytokines, immunocyte infiltrates) was evident in the kidneys of C. albicans-infected and C. parapsilosis-infected Syk- or CARD9-deficient mice at 2 and 30 dpi, respectively, it likely does not contradict the decreased proinflammatory cytokine production of Syk^−/−^ or CARD9^−/−^ myeloid cells but is, rather, the consequence of unrestricted fungal presence triggering Syk/CARD9-independent signaling (e.g., TLR-MyD88 pathways). In the case of the C. parapsilosis challenge, immunocyte infiltrates and elevated amounts of proinflammatory cytokines were more characteristic in the kidneys of Syk^−/−^ animals than in those of CARD9^−/−^ mice at 30 dpi. This may be the consequence of the CARD9-dependent, but Syk-independent, chemokine production observed in C. parapsilosis-treated macrophages that could allow for more efficacious leukocyte recruitment to the site of infection in Syk^−/−^ chimeras than in CARD9^−/−^ chimeras.

The mechanism through which Syk and CARD9 counteract C. albicans infections is grounded in signaling initialized by Dectin-1, Dectin-2, Dectin-3, Mincle, and CR3 (12, 59–63). However, it is unclear which receptors utilize the Syk/CARD9 pathway to provide the observed *in vivo* protection against C. parapsilosis. Cell culture experiments with C. parapsilosis revealed that Dectin-1 could regulate immune responses *in vitro* (10, 12, 42). In contrast, phagocytosis of C. parapsilosis by human neutrophils was independent of Dectin-1 (64). Furthermore, no dependence on Dectin-1 in long-term (21 dpi) clearance of C. parapsilosis was reported (12), and our previous results demonstrated that the fungal load was unaffected by Dectin-1 using several time points (1, 3, 7, 14, and 20 dpi) and multiple C. parapsilosis strains (65). Therefore, it is tempting to hypothesize the involvement of Syk/CARD9-bound PRRs other than Dectin-1 in systemic resistance against C. parapsilosis. Fitting with the crucial role of Syk in B-cell development (66, 67) and that Syk was involved in more cellular processes in response to *Candida* stimuli than CARD9 in our *in vitro* experiments, fungal burdens in Syk^−/−^ chimeras tended to surpass those of CARD9^−/−^ chimeras. Therefore, we propose that Syk has a more prominent function in antifungal immunity than CARD9 against both *Candida* species.

Notably, the excess of fungal burden in some organs (kidneys, livers, brain) of C. parapsilosis-challenged Syk^−/−^ or CARD9^−/−^ chimeras only surpassed that of WT chimeras dramatically at 30 dpi and, by this time, leukocyte infiltrates containing macrophages, neutrophils, and T cells and proinflammatory cytokine production were also evident in the kidneys of mostly Syk^−/−^ chimeras. In contrast, the kidneys and brain of Syk^−/−^ and the kidneys of CARD9^−/−^ chimeras treated with C. albicans were marked by more than 100-fold higher colonization and massive inflammation by 2 dpi. This is well before adaptive immunity could develop. Regarding systemic resistance, this may suggest that the Syk/CARD9 pathway plays a greater role in innate immunity against C. albicans than C. parapsilosis. In our setting, it appears that Syk- and CARD9-independent innate mechanisms initially exert some control over the growth of C. parapsilosis in host tissues. However, it is possible that the absence of Syk and CARD9 is sufficient for a failure in the development of adaptive immunity, and innate mechanisms alone are unable to prevent the proliferation of host-adapted C. parapsilosis cells over an extended period of infection. The notion that both proper innate and adaptive immunity are required for the total elimination of this yeast is corroborated by our observation that severe combined immunodeficient mice—infected according to the same methods as this study—were unable to clear C. parapsilosis as efficiently as WT mice (unpublished data). The species-specific kinetic patterns of fungal clearance by the Syk^−/−^ or CARD9^−/−^ chimeras may also arise from inherent differences between C. parapsilosis and C. albicans. For example, a study found less beta-glucan in the cell wall of C. parapsilosis than that of C. albicans (12). Accordingly, while the beta-glucan receptor Dectin-1 can play a crucial role in the resistance against C. albicans (12, 59, 68), it seems less important against C. parapsilosis (12, 65). In the absence of Syk or CARD9, Dectin-1 signaling is damaged, promoting rapid initial expansion of C. albicans yeast cells in the host. Subsequently, the formation of more virulent hyphae faces only limited control without signaling from Dectin-2 and Dectin-3 (23, 62), allowing for fast overgrowth as demonstrated in the kidneys of Syk^−/−^ or CARD9^−/−^ chimeras at 2 dpi. Extensive hyphal colonies of C. albicans comprise a potent inducer of inflammation through mechanisms such as the production of the candidalysin toxin that induces host cell lysis (69). In contrast, C. parapsilosis does not synthesize candidalysin nor form hyphae (7), which leaves it prone to phagocytosis throughout the whole duration of infection. Additionally, this yeast is less capable of triggering danger signals or inflammation (7, 38) and can actively hinder inflammatory responses by enhancing IL-27 signaling (11). For example, IL-27 may downregulate IL-17 signaling and therefore attenuate neutrophil functions (11, 70, 71). All of these may contribute to the gradual overgrowth of C. parapsilosis that eventually induces inflammatory responses as observed at 30 dpi in the Syk^−/−^ or CARD9^−/−^ chimeras. Delayed inflammation in an immunocompromised host, such as the Syk^−/−^ or CARD9^−/−^ chimeras, may serve to grant C. parapsilosis a prolonged window for proliferation rather than leading to swift death. As, unlike C. albicans, C. parapsilosis is horizontally transmissible (7, 72); this strategy may allow for exploiting the host as a reservoir for propagation.

Before this study, directly Syk-dependent immunity to a nonalbicans *Candida* species in an *in vivo* model was not reported. Recent investigations demonstrated that Syk mediates key neutrophil responses to C. albicans, C. glabrata, and C. auris *in vitro*. Based on their data, the authors suggested that “Syk may have differential roles depending on the fungal species” (46). In accordance, our study is the first to show that Syk may regulate protective immune responses to *Candida* infections species-specifically *in vivo*. CARD9 is also differentially involved in immunity to *Candida* species (36), which our data confirm. Additionally, our study offers a methodological novelty through the use of chimeras fully devoid of Syk or CARD9 selectively in their hematopoietic systems to examine anti-*Candida* immune responses. The use of chimeras was necessitated by the perinatal mortality of fully Syk knockout (KO) mice (66, 67); therefore, we opted for CARD9^−/−^ bone marrow chimeras instead of fully CARD9 KO animals for better comparison. Using this model, phenotypes are better attributable to immunocytes than a fully KO model would allow, as the expression of Syk/CARD9-dependent receptors is not restricted to immunocytes (73, 74).

Our work prompts further investigations on exactly which PRRs and cell types are responsible for Syk/CARD9-mediated functions in response to C. parapsilosis. While Syk/CARD9 signaling confers protection against C. albicans largely via neutrophils (26, 33, 46), our ongoing research challenges the assumption that the susceptible phenotype of Syk^−/−^ or CARD9^−/−^ chimeras infected with C. parapsilosis is a result of compromised neutrophil functions (data not shown). Dissection of how this pathway regulates adaptive immunity to C. parapsilosis and understanding on a molecular level of how CARD9 may operate independently of Syk in myeloid cells must also be achieved in the future. Finally, testing Syk and CARD9 agonists in the setting of experimental infections by common pathogenic *Candida* species will provide valuable information on the curative prospects of Syk/CARD9 signaling on a species-specific scale.

Over the last decade, the modulation of the Syk/CARD9-dependent mechanisms has been proposed as an approach to combat microbial infections (75), and the regulation of Syk activity for therapeutic purposes has become reality (76). Our findings support ongoing efforts to target this pathway for anti-*Candida* immune therapy.

## MATERIALS AND METHODS

### Ethics statement.

Animal experiments were carried out in accordance with the Hungarian national (1998.XXVIII; 40/2013) and European (2010/63/EU) animal ethics guidelines. Procedures were approved by the Animal Welfare Committees of the University of Szeged and the Semmelweis University as well as the Hungarian National Animal Experimentation and Ethics Board. The license numbers for animal experiments performed in this work were XIV‐I-001/2150-4/2012 for the generation of bone marrow chimeras and XVI./3646/2016 with the modification CSI/01/3646-6/2016 for the *in vivo* candidiasis experiments.

### Mice.

Syk-deficient, CARD9-deficient, and the respective wild-type bone marrow chimeric mice (referred to as Syk^−/−^, Wt_Syk_, CARD9^−/−^, and Wt_CARD9_ bone marrow chimeras) were used in this study for the cultivation of macrophages and for *in vivo* experiments.

Syk^−/−^ and Wt_Syk_ bone marrow chimeric mice were generated by fetal liver transplantation as described previously (77) with minor modifications. Briefly, heterozygous mice on the C57BL/6 genetic background harboring a deleted Syk allele (*Syk*^tm1Tyb^) (66) were mated. Syk^−/−^ fetuses were distinguished based on their petechiae morphology along with PCR (78) and used to obtain Syk^−/−^ bone marrow chimeras. Fetuses with normal morphology (Syk^+/+^ and the Syk^+/−^ genotypes) were utilized for the generation of Wt_Syk_ chimeras. Embryos of 17 to 18 days were used to isolate fetal liver cells. The recipient mice (∼8 to 10 weeks old) on the C57BL/6 genetic background were lethally irradiated by 11 Gy from a ^137^Cs source and subsequently injected intravenously with fetal liver cell suspensions. Syk deficiency was checked 4 weeks after the transplantation based on the defective B-cell differentiation in Syk-deficient hematopoietic systems (66). Blood was sampled and stained with anti-B220 (clone RA3-6B2), anti-Ly6G (clone 1A8), and anti-CD45.2 (clone 104) (all from BD Biosciences) antibodies for flow cytometric analysis. Bone marrow chimeras were considered Syk^−/−^ if, during the time of detecting 500-neutrophil granulocytes (CD45.2^+^Ly6G^+^ cells), the proportion of B cells (CD45.2^+^ B220^+^ cells) was no more than 8% within the total population consisting of B cells and neutrophils together.

Bone marrow transplantation was applied in order to generate CARD9^−/−^ and Wt_CARD9_ bone marrow chimeras as described (79). Wild-type and CARD9^−/−^ C57BL/6 mice [*Card9*^tm1a(EUCOMM)Hmgu^] homozygous for the CD45.2 allele served as bone marrow donors, and a congenic strain carrying the CD45.1 allele on the C57BL/6 genetic background (B6.SJL-Ptprc^a^) was used as recipient. Bone marrow cells were intravenously injected into previously lethally irradiated recipients (∼8 to 20 weeks old). Four weeks later, peripheral blood was stained with anti-Ly6G and anti-CD45.2 antibodies (both from BD Biosciences) and assessed by flow cytometry. The transplantation was considered sufficient if over 98% of Ly6G^+^ neutrophils were CD45.2^+^.

### Cell cultures.

The macrophage colony-stimulating factor (M-CSF)-producing L929 fibroblast cell line (a kind gift from Csaba Vizler, Biological Research Centre, Szeged) was used to obtain L929-conditioned medium. To this end, confluent cultures were incubated with nonsupplemented Dulbecco’s modified Eagle’s medium (DMEM; Lonza) in 75-ml tissue culture flasks for 10 days. Cell culture supernatants were subsequently sterile filtered, aliquoted, and kept at −20°C until utilization.

Based on previous studies (25, 80), primary bone marrow-derived macrophages (BMDMs) were cultured from the bone marrows of 8- to 15-week-old female and male bone marrow chimeric mice in BMDM medium (80% [vol/vol] DMEM supplemented with 10% heat-inactivated fetal bovine serum [FBS; Lonza] and 1% penicillin-streptomycin mixture [Lonza]; 20% [vol/vol] L929-conditioned medium) for 7 to 9 days in 96-, 24-, or 12-well plates, according to the experiment. Fresh medium was added every other day.

We checked the functionality of the macrophage culturing method by immunostaining with anti-CD11b (Sony) and anti-F4/80 antibodies (BioLegend) or isotype controls (Sony and BioLegend) followed by flow cytometric analysis where CD11b^+^ F4/80^+^ double-positive cells were considered macrophages (81). As the proportion of these cells was over 80% in the case of all genotypes (data not shown), we regarded the method as functional.

### Fungal strains and preparation for experiments.

C. parapsilosis GA1 (SZMC 8110), CLIB214 (SZMC 1560), CDC317, and C. albicans SC5314 (SZMC 1523) clinical isolates and a GFP-expressing derivative of CLIB214 (genotype, CpNEUT5L/CpNEUT5L::pECpOE-GFP-N-N5L) were used in this study. All strains were maintained on YPD agar plates (1% yeast extract, 2% bactopeptone, 2% glucose, and 2% agar) at 4°C and were refreshed monthly by streaking onto fresh medium followed by 2 days of incubation at 30°C. Before experiments, *Candida* cells were grown overnight at 30°C in 2 ml liquid YPD medium (no agar), and 200 μl of the suspension was added to another 2 ml of liquid YPD for a second round of overnight incubation at 30°C. Yeast cells were harvested by centrifugation, washed three times with phosphate-buffered saline (PBS), and counted using a hemocytometer. Adequate cell concentrations determined by the multiplicity of infection (MOI) for the individual experiments were set in PBS for *in vivo* infection experiments or in DMEM supplemented with 10% heat-inactivated FBS and 1% penicillin-streptomycin mixture for *in vitro* coincubation experiments.

### Nuclear translocation of NF-κB p65.

Macrophages were infected with C. parapsilosis (MOI of 5:1) or treated with LPS (1 μg/ml, positive control) in 12-well plates for 90 min. Cell culture medium was refreshed on untreated control cells. At 75 min, DRAQ5 (Thermo Fisher Scientific) nuclear stain (2.5 μM) was added to each well, and the incubation was continued for 15 min. Supernatants were discarded, and wells were washed twice with PBS. Macrophages were trypsinized with TrypLE Express enzyme (Gibco) for 5 min and were suspended with 10% FBS-PBS. Staining of NF-κB p65 was carried out based on an R&D Systems protocol (https://www.rndsystems.com/resources/protocols/flow-cytometry-protocol-staining-intracellular-molecules-using-detergents). Briefly, centrifuged macrophages were fixed in 4% PFA-PBS and washed twice. Cells were suspended in 100 μl 0.3% Triton X-100-PBS and were stained with 5 μl Alexa Fluor 488-NF-κB p65 antibody (R&D Systems) for 30 min in the dark. After two further washing steps, macrophages were loaded in the Amnis FlowSight imaging flow cytometer in 100 μl PBS. Brightfield and fluorescence microscopic images of single macrophages were captured using laser excitation at 488 and 642 nm. Nuclear Localization Wizard of the IDEAS 6.2 software was utilized to determine cell populations with NF-κB p65 translocated into nuclei.

### Detection of cytokines by Proteome Profiler.

BMDMs were infected with C. parapsilosis (GA1 strain; MOI of 5:1) for 24 h in 24-well plates, and cell culture supernatants were pooled from at least 3 independent experiments. The Proteome Profiler mouse cytokine array panel A kit (R&D Systems) was applied for multiplex detection of cytokines according to the manufacturer’s instructions (https://resources.rndsystems.com/pdfs/datasheets/ary006.pdf). A total of 700 μl pooled supernatant was used for a single membrane. Chemiluminescence was visualized by Image Studio Digits 3.1.

### Measurement of *in vitro* cytokine production by ELISA.

BMDMs were cultured in 24-well plates and infected with C. parapsilosis strains (MOI of 5:1) or the C. albicans strain (MOI of 1:25) for 24 h. The concentrations of TNF-α, KC, MIP1-α, and MIP-2 in cell culture supernatants were determined by commercial ELISA kits (R&D Systems; catalog nos. DY410, DY453, DY450, and DY452, respectively) in accordance with the manufacturer’s instructions. OD measurement was carried out using the SPECTROstar Nano microplate reader (BMG Labtech), and data were analyzed with the MARS data analysis software.

### Phagocytosis assay.

The experiment was carried out as described (38, 82). In short, yeast cells stained with Alexa Fluor 488 succinimidyl ester (Invitrogen) or a GFP-expressing transformant derived from the CLIB214 C. parapsilosis isolate were cocultured with macrophages (MOI of 5:1) in 12-well cell culture plates. In the case of C. parapsilosis strains, coincubation times were 15 min and 120 min. As hyphae formation may interfere with phagocytosis and hinder the quantification of ingested yeast cells, 15-min and 30-min time points were applied for C. albicans infections to avoid hyphenation. Macrophages were washed twice with PBS, trypsinized, and suspended in FBS-PBS. Pelleted cells were resuspended in 100 μl PBS and loaded into the Amnis FlowSight imaging flow cytometer. Singlet macrophages were gated and monitored with white light and 488 nm laser illumination. The IDEAS 6.2 software was used for data analysis. Alexa Fluor 488^+^ or GFP^+^ macrophages were defined as the phagocytosing cell population. Microphotographs of individual macrophages were submitted to the Spot Count Wizard to determine the average number of ingested yeast cells per macrophage within the phagocytosing population.

### Phagosome acidification.

The method used by Papp et al. (83) was modified in this experiment. *Candida* cells were dually labeled with Alexa Fluor 488 succinimidyl ester and the pH-sensitive fluorescent dye pHrodo Red succinimidyl ester (Invitrogen) in Hanks’ balanced salt solution. The GFP-expressing CLIB214 strain was only stained with pHrodo Red succinimidyl ester. Macrophages were infected with the labeled yeast cells (MOI of 5:1) in 12-well cell culture plates for 15 min. Cocultures were then treated and loaded into the Amnis FlowSight imaging flow cytometer as in the phagocytosis assay. Singlet macrophages were examined with white light and 488 nm laser illumination. A compensation matrix was applied to eliminate the partially overlapping emission of pHrodo Red and Alexa Fluor 488 or GFP. We used the IDEAS 6.2 software for data analysis. While pHrodo Red gains bright fluorescence in acidified phagosomes, the emission of Alexa Fluor 488 is independent of cellular localization. Thus, phagosome acidification efficacy was calculated as follows:

$$\frac{{\text{proportion of pHrodo Red}}^{+}\text{macrophages}}{{\text{proportion of Alexa Fluor 488}}^{+}{\text{or GFP}}^{+}\text{macrophages}}\times 100\%$$

### Elimination of C. parapsilosis by macrophages.

Macrophages were coincubated with different C. parapsilosis strains (MOI of 5:1) in 24-well plates in triplicates for 3 h. Macrophage-free control wells with identical numbers of yeast cells were also used. After incubation, macrophages were disrupted by adding distilled water and forcibly pulling the culture through a 26-gauge needle 5 times. Serially diluted lysates were plated on YPD plates and incubated at 30°C for 2 days. The number of CFU was determined, the average values of replicates were calculated, and the killing efficiency was determined as follows:

$$\frac{{\text{CFU}}_{\text{control well}}–{\text{CFU}}_{\text{coculture well}}}{{\text{CFU}}_{\text{control well}}}\times 100\%$$

### *In vivo* infection of bone marrow chimeras and fungal burden of tissues.

Mice were injected with C. parapsilosis (2 × 10^7^ yeast cells/100 μl PBS per mouse) or C. albicans (2 × 10^5^ yeast cells/100 μl PBS per mouse) via the lateral tail vein. C. parapsilosis-infected animals were euthanized 2, 5, 7, and 30 dpi, while C. albicans-infected ones were sacrificed on day 2. Blood was sampled from the retro-orbital plexus or the caudal *vena cava* and was plated on YPD agar plates. Spleens, kidneys, livers, and brains were surgically collected, weighed, and homogenized in 2 ml PBS using a TT-30K digital handheld homogenizer (Hercuvan). Homogenates were plated on YPD agar plates. CFU were counted after 2 days of incubation at 30°C, and fungal burden was calculated as CFU/ml blood or CFU/g tissue for the assessed organs.

### Cytokine content in kidneys.

Mice were infected with C. parapsilosis or C. albicans as described in the previous paragraph or mock infected with 100 μl PBS. Kidneys were harvested 2 and 30 (in the case of C. parapsilosis) dpi and were homogenized. The LEGENDplex bead-based immunoassay approach (BioLegend) was used for multiplex identification of cytokines in the kidney homogenates using a BD FACSAria Fusion device (BD Biosciences) and the BD FACSDiva software.

### Histopathology of kidneys.

Mice were infected with C. parapsilosis or C. albicans as described above. Kidneys were collected and fixed in 4% paraformaldehyde (PFA)-PBS 2 and 30 (in the case of C. parapsilosis) dpi, and periodic acid-Schiff (PAS)-stained sections were prepared to detect fungal elements and leukocyte infiltrates. Preparations labeled with rabbit anti-MPO (Abcam), anti-CD68, and anti-CD3 (Boster Biological) primary antibodies and stained with kits implementing anti-rabbit secondary antibodies (Abcam, catalog no. ab209101; Boster Biological, catalog no. SV0002-1) were also made to visualize the presence of neutrophil granulocytes, macrophages, and T cells, respectively.

### Quantification and statistical analysis.

Significance was determined at *P* < 0.05. All *in vitro* assays were analyzed by paired Student's *t* test. Mann-Whitney tests were used for the evaluation of fungal burden, and two-way analysis of variance (ANOVA) was applied for the assessment of the kidney cytokine content. Tests were performed and diagrams were created with the GraphPad Prism 6.0 software. Statistical details and tests are shown in the figure legends.
