# Supplementary figures and images for: Signaling through Syk or CARD9 Mediates Species-Specific Anti-Candida Protection in Bone Marrow Chimeric Mice
Source: mBio. 2021 Aug 31;12(4):e01608-21. doi: 10.1128/mBio.01608-21 (PMC8406149; doi:10.1128/mBio.01608-21)

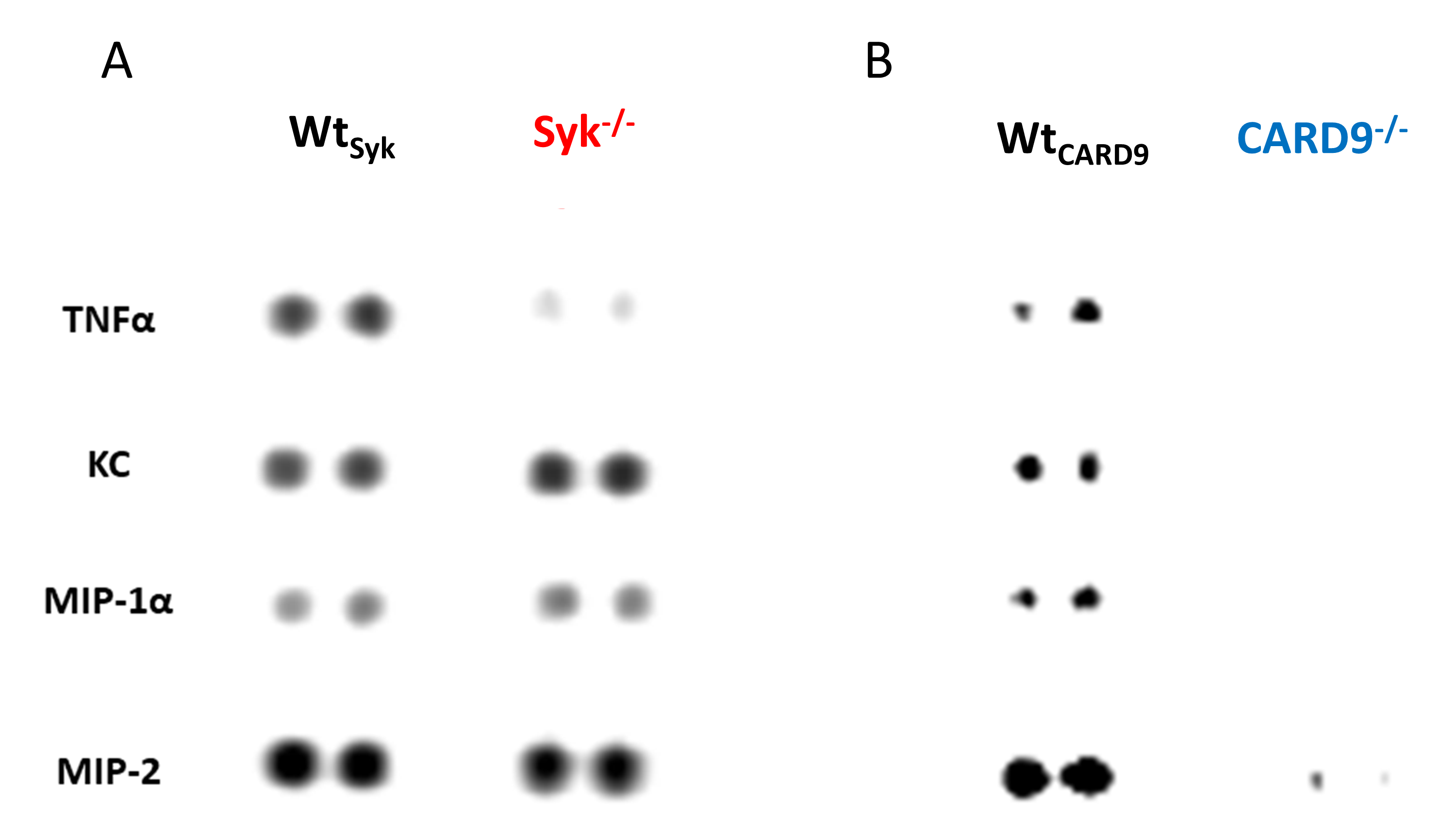

Supplement: FIG S1 [file mbio.01608-21-sf001.tif]

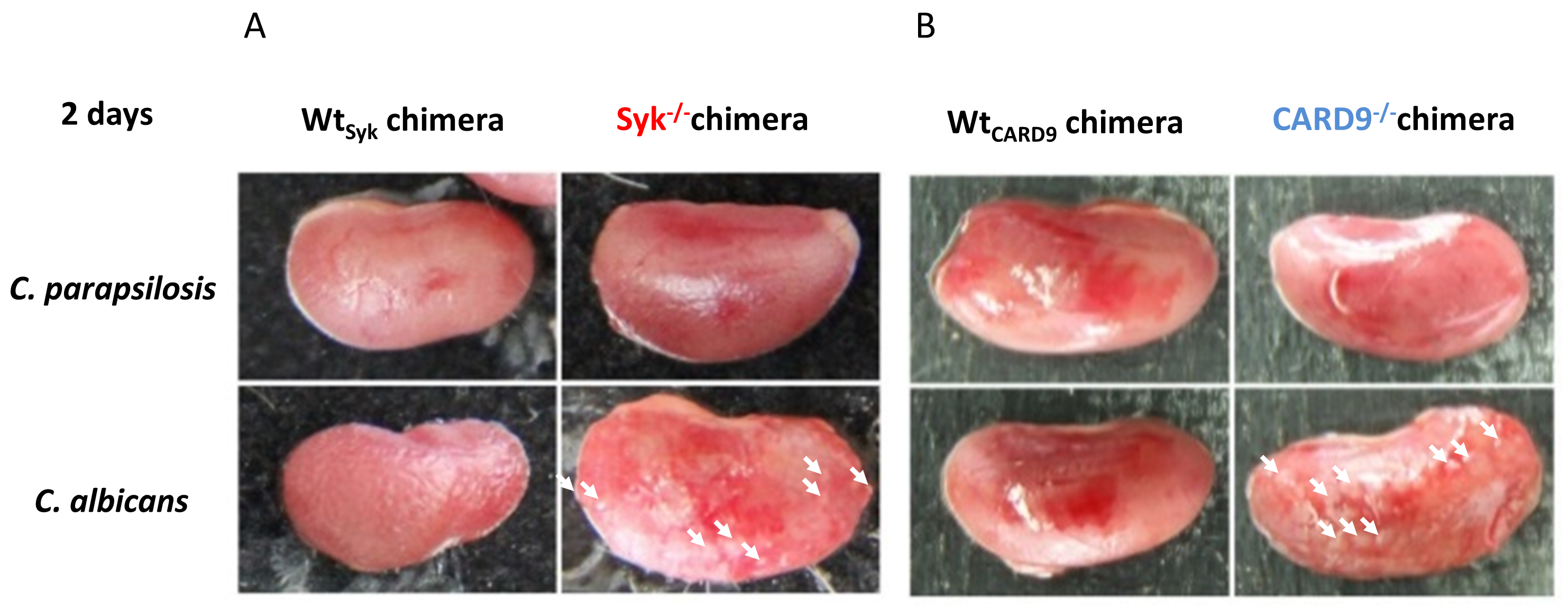

Supplement: FIG S3 [file mbio.01608-21-sf003.tif]
