# Supplementary material for: Signaling through Syk or CARD9 Mediates Species-Specific Anti-Candida Protection in Bone Marrow Chimeric Mice
Source: mBio. 2021 Aug 31;12(4):e01608-21. doi: 10.1128/mBio.01608-21 (PMC8406149; doi:10.1128/mBio.01608-21)
Supplement: TABLE S1 [file mbio.01608-21-st001.pdf]

TABLE S1. Absolute cytokine concentrations (pg/ml) ± SD measured by ELISA from cell culture supernatants of BMDMs treated with *C. parapsilosis* (strains GA1, CLIB214 or CDC317, MOI 5:1, 24 h) or *C. albicans* (strain SC5314, MOI 1:25, 24 h). Data are pooled from a minimum of 4 independent experiments. ctr: control, Cp-: *C. parapsilosis*; Ca-: *C. albicans*.

	Wt_{Syk}					$Syk^{-/-}$				
	**ctr**	**Cp-GA1**	**Cp-CLIB214**	**Cp-CDC317**	**Ca-SC5314**	**ctr**	**Cp-GA1**	**Cp-CLIB214**	**Cp-CDC317**	**Ca-SC5314**
**TNFα**	5.2±11.7	679.7±553.7	2068.7±1757.4	164.7±72.5	1756.7±1395.5	0±0	370±235	758.3±537	15.4±12.4	23.1±51.7
**KC**	30.1±43.6	1488.4±1331.9	2414.9±1841	429.7±245.7	1324.4±553.5	35±61.1	2149.1±2722.4	2631.7±2923	394.9±390	103.3±177.5
**MIP-1α**	32.8±24.8	864.8±704.9	1121.6±748.2	131±128.4	1143.1±464.8	27.7±17.6	810.6±826.4	1091.7±741.1	64.2±40	119±57.1
**MIP-2**	209±79.5	6840±2255.7	9447.2±2310.2	2301.3±227.4	12787.6±3967.5	162±96.7	7260.7±4220.8	8696.3±3920.8	1742.6±846.8	1021.362±1428.8
	WT_{CARD9}					$CARD9^{-/-}$				
	**ctr**	**Cp-GA1**	**Cp-CLIB214**	**Cp-CDC317**	**Ca-SC5314**	**ctr**	**Cp-GA1**	**Cp-CLIB214**	**Cp-CDC317**	**Ca-SC5314**
**TNFα**	92.7±160.5	217.8±178.2	656.5±501.4	285.2±317	207.3±192.3	66.6±108	105.8±186.2	379.6±288.1	137±174.1	93.7±166.6
**KC**	1.8±4.4	685.6±555.5	1531.7±916.1	334.8±287	322.8±214.3	7.6±18.6	206.4±239.9	931.9±606.7	224.5±249.9	0.8±2.2
**MIP-1α**	60.7±70.9	674.8±668.8	1126±626.7	279.8±330.9	478±342.1	33.5±47.9	275.9±362.3	716.6±529.2	115.8±132.1	147.4±217.4
**MIP-2**	0±0	1835.4±562.5	4125.7±911	1518.6±750.7	3037.2±737	46.7±57.8	766.7±197.2	2358.8±649.6	845.6±529.7	115±85.4
